# Serotonin Receptor 2C and Insulin Secretion

**DOI:** 10.1371/journal.pone.0054250

**Published:** 2013-01-17

**Authors:** Qiang Zhang, Yunxia Zhu, Wenbo Zhou, Lu Gao, Li Yuan, Xiao Han

**Affiliations:** State Key Laboratory of Reproductive Medicine and Key Laboratory of Human Functional Genomics of Jiangsu Province, Nanjing Medical University, Nanjing, Jiangsu, China; CRCHUM-Montreal Diabetes Research Center, Canada

## Abstract

Type 2 diabetes mellitus (T2DM) describes a group of metabolic disorders characterized by defects in insulin secretion and insulin sensitivity. Insulin secretion from pancreatic β-cells is an important factor in the etiology of T2DM, though the complex regulation and mechanisms of insulin secretion from β-cells remains to be fully elucidated. High plasma levels of serotonin (5-hydroxytryptamine; 5-HT) have been reported in T2DM patients, though the potential effect on insulin secretion is unclear. However, it is known that the 5-HT receptor 2C (5-HT_2C_R) agonist, mCPP, decreases plasma insulin concentration in mice. As such, we aimed to investigate the expression of the 5-HT_2C_R in pancreatic islets of diabetic mice and the role of 5-HT_2C_R signaling in insulin secretion from pancreatic β-cells. We found that 5-HT_2C_R expression was significantly increased in pancreatic islets of db/db mice. Furthermore, treatment with a 5-HT_2C_R antagonist (SB242084) increased insulin secretion from pancreatic islets isolated from db/db mice in a dose-dependent manner, but had no effect in islets from control mice. The effect of a 5-HT_2C_R agonist (mCPP) and antagonist (SB242084) were further studied in isolated pancreatic islets from mice and Min-6 cells. We found that mCPP significantly inhibited insulin secretion in Min-6 cells and isolated islets in a dose-dependent manner, which could be reversed by SB242084 or RNA interference against 5-HT_2C_R. We also treated Min-6 cells with palmitic acid for 24 h, and found that the expression of 5-HT_2C_R increased in a dose-dependent manner; furthermore, the inhibition of insulin secretion in Min-6 cells induced by palmitic acid could be reversed by SB242084 or RNA interference against 5-HT_2C_R. Taken together, our data suggests that increased expression of 5-HT_2C_R in pancreatic β-cells might inhibit insulin secretion. This unique observation increases our understanding of T2DM and suggests new avenues for potential treatment.

## Introduction

Type 2 diabetes mellitus (T2DM) is a chronic metabolic syndrome caused by insulin deficiency [1]. T2DM patients usually display loss of insulin sensitivity in white adipose tissue, skeletal muscle and liver, accompanied by disorders of insulin secretion [2]. Insulin is secreted from pancreatic β-cells and is controlled by blood glucose level; low blood glucose level induces basal insulin secretion whereas high blood glucose levels, as encountered postprandially, will increase insulin secretion up to five to ten times [3]. In addition to blood glucose, lipids, cytokines and hormones can all affect insulin secretion, as such the mechanisms underlying the dysfunction of pancreatic β-cells in T2DM patients is complex and is far from being fully understood [2].

As far as we know, the pancreatic islet is highly innervated by parasympathetic and sympathetic neurons [4]. Recent studies have demonstrated that the nervous system can regulate insulin secretion from pancreatic β-cells through neurotransmitters and their respective receptors expressed in β-cells [5]. For example, the cholinergic nerves [6] and the sympathoadrenal axis [7] can modulate insulin secretion from pancreatic β-cells, demonstrating that the nervous system can regulate insulin secretion directly, and in addition to indirect effects on metabolism through the regulation of food intake, body temperature, sleep and activity [8]. There is evidence that the pancreas receives serotonergic nervous inputs from vagus and enteric nervous system [9]. The serotonin (5-hydroxytryptamine; 5-HT) secreted form these intrapancreatic nerves may act as a stimulator or inhibitor of pancreatic exocrine secretion, depending on the expression of different receptor subtypes [9], but whether the 5-HT system could play a role in pancreatic endocrine function is largely unknown [10], [11]. Furthermore, it has been suggested that pancreatic β-cells can secrete 5-HT by themselves, which could represent a form of autocrine regulation [12].

5-HT is a biogenic amine that is synthesized in the enteric nervous system and the central nervous system [13]; it has a wide variety of physiological functions, including regulating body temperature, cardiovascular function, mood, bodyweight, and cognitive functions [14]. Recent studies have demonstrated that the 5-HT system could be involved in glucose and lipid metabolism [15], [16], as well as adipocyte differentiation [17]. The 5-HT receptors, excluding 5-HT receptor 3, are all members of the GPCR superfamily of signal transducing receptors [18]. The 5-HT receptor 2C (5-HT_2C_R) is through to be the most critical 5-HT receptor for regulating energy homeostasis [19], [20], [21]. The predominant function of signaling through the 5-HT_2C_R is thought to drive the anorexic effect in hypothalamus [22]; however there are also reports that 5-HT is increased in the plasma and brains of diabetic patients and hyperphagic people [23], [24]. Moreover, chronic hyperphagia of Ay mice increases the expression of 5-HT_2C_R in the hypothalamus, demonstrating that the function of the 5-HT system could be enhanced in diabetic and obese individuals [25]. Interestingly, Zhou et al. found that after 14 days of treatment with mCPP (a 5-HT_2C_R agonist), mice with diet-induced obesity exhibited reduced circulating insulin concentrations, while blood glucose, body weight, and feeding remained unchanged [26]. This data suggests a direct effect of mCPP in decreasing insulin secretion through activating 5-HT_2C_R.

It is known that T2DM patients usually display delayed elevation of plasma concentration of insulin postprandially [27], which is when the vagus and enteric neurons are highly activated, resulting in increased circulating levels of 5-HT [28]. As such, our first aim was to study the expression of 5-HT_2C_R in pancreatic islets from diabetic mice, and then study its effect on insulin secretion. Given that patients with T2DM usually have hyperlipidemia, which can cause insulin resistance and decreased insulin secretion [29], our second aim was to investigate the effect of palmitic acid on the expression of 5-HT_2C_R in pancreatic β-cells.

## Materials and Methods

### Reagents

The 5-HT_2C_R agonist, mCPP (1-(3-chlorophenyl)piperazine hydrochloride) was purchased from Sigma (Sigma-Aldrich Co., St Louis, MO), and the 5-HT_2C_R antagonist, SB242084, (2HCl(6-chloro-5-methyl-1-[6-(2-methylpyridin-3-yloxy) pyridin-3-ylcarbamoyl] indo-line) was purchased from Tocris (Tocris Bioscience, Bristol, UK). Palmitic acid was purchased from Sigma (Sigma–Aldrich), Dulbecco’s modified Eagle media (DMEM), RPMI 1640 medium and fetal bovine serum (FBS) were all purchased from Gibco (Life Technologies Co., Grand Island, NY). The antibodies against 5-HT_2C_R were purchased from Santa Cruz Biotechnology (Santa Cruz Biotechnology, Santa Cruz, CA). The antibodies against THP1 and THP2 were purchased from Bioss (Bioss, Massachusetts, USA). TaqMan one-step PCR kit and Assays-on-Demand gene expression products were purchased from ABI (Applied Biosystems, Life Technologies Co.).

### Cell Culture

Min-6 cells (passage 20 to 30) were grown in DMEM medium containing 15% FBS, 25 mmol/l glucose, 50 µmol/l 2-mercaptoethanol, 100 U/ml penicillin and 100 µg/ml streptomycin. The cells were cultured at 37°C in a humidified atmosphere containing 95% air and 5% CO2. For all compounds prepared in DMSO and ethanol, the final concentration in the culture medium was kept at less than 0.2%.

### Islet Purification and Culturing

All animal studies were performed according to guidelines established by the Research Animal Care Committee of Nanjing Medical University. Male db/db mice (17 weeks of age; C57BL/KsJ; n = 6 per group) and control C57BL/KsJ mice were purchased from the Shanghai Institute of Materia Medica, Chinese Academy of Sciences. Male ICR mice (23 to 25 g body weight) were purchased from Nanjing Medical University Laboratory Animal Centre, Nanjing, China. Islet isolation and culturing techniques have been described previously [30]. Freshly isolated islets were transferred to sterile six-well dishes and cultured in 1640 medium containing 11.1 mmol/l glucose supplemented with 10% FBS, 10 mmol/l HEPES, 100 U/ml penicillin and 100 µg/ml streptomycin. The islets were allowed to equilibrate for 3 h, after which they were counted and repicked into six well plates (400 islets per well for RNA or protein extraction) or 48 well plates (8 islets per well for glucose stimulated insulin secretion [GSIS]) and cultured overnight at 37°C for future analysis.

### Real Time Reverse Transcription-polymerase Chain Reaction

Total RNA of primary mouse islets and Min-6 cells were extracted by TRIzol reagent (Invitrogen, Life Technologies Co.), according to the manufacturer’s protocol. After quantification by spectrophotometry, 1 µg of total RNA was used for reverse transcription in a final volume of 20 µl with AMV Reverse Transcriptase (Promega, Madison, WI, USA), according to the manufacturer’s instructions. Aliquots of cDNA corresponding to equal amounts of RNA were used for the quantification of mRNA by real-time PCR using the ABI Prism 7000 Sequence Detection System (Applied Biosystems, Life Technologies Co.). The reaction mixture contained the corresponding cDNA, forward and reverse primers, and SYBR green PCR Master Mix (Applied Biosystems, Life Technologies Co.). Relative expression of the different gene transcripts was calculated by the 2^ΔΔCt^ method. The specific primers were as follows: 5-HT_2C_R forward: 5′-GTT CAA TTC GCG GAC TAA GG-3′, reverse: 5′-TCA CGA ACA CTT TGC TTT CG-3′; THP1 forward: 5′-GAC CAT CTT CCG AGA GCT AAA CAA-3′, reverse: 5′-AGC AAA GGG AGG TTT CTG AGG TA-3′; THP2 forward: 5′-ATG TGG CAA AAC GGA ATT CAA T-3′, reverse: -5′-CCT CCG TCC AAA TGC TCT CA-3′; β-actin forward: 5′-CAG ACA ACA TAA ACT GCG CCT TT-3′, reverse: 5′-GGA TAC ACC TCT CCA CCA ATG AC-3′. All gene expression data was analyzed using β-actin gene expression as a reference gene.

### Western Blot Analysis

To isolate protein, cells were lysed in an ice-cold lysis buffer containing 50 mM Tris-HCl (pH 7.4), 1% NP-40, 150 mM NaCl, 1 mM EDTA, 1 mM phenylmethylsulfonyl fluoride, and a complete proteinase inhibitor cocktail (Pierce, Rockford, IL). Protein concentration in the cell lysate was quantified using the DC protein assay kit (Bio-Rad, Hercules, CA.). An equal amount of protein (20 µg) for each sample was separated by 10% SDS-PAGE and transferred to PVDF membranes (Millipore, Billerica, MA.). After blocking, the membranes were incubated at 4°C overnight with the respective antibodies against 5-HT_2C_R (diluted 1∶1,000), TPH1 (diluted 1∶300), TPH2 (diluted 1∶300) or β-actin (1∶4,000), in 1% bovine serum albumin in TBST buffer. Subsequently, the blots were washed in TBST buffer and then incubated with horseradish peroxidase-conjugated anti-goat (diluted 1∶10,000), anti-rabit (diluted 1∶500) or anti-mouse (diluted 1∶4,000) secondary antibodies for 1 h at room temperature. Protein bands were visualized using enhanced chemiluminescence reagents (ECL Plus Detection System; Amersham Pharmacia Biotech, Orsay, France). Prestained markers were used as internal molecular weight standards. The gray scale values of the bands were calculated using ImageJ software (free downloaded from NIH website: http://rsbweb.nih.gov/ij/).

### RNA Interference

The 5-HT_2C_R siRNAs targeted three regions of 5-HT_2C_R mRNA for interference, and were purchased from RuiBo company (Guangzhou, China). The target sequences were as follows: si5-HT_2C_R-1: 5′-GCA CAA UGC UAC CAA UUA U dTdT-3′; si5-HT_2C_R-2: 5′-CGU CGA AAG AAG AAA GAA A dTdT-3′; si5-HT_2C_R-3: 5′-CUA UCA ACA AUG AGA AGA A dTdT-3′. A control siRNA was used in the control group. The control siRNA was also purchased from RuiBo company (Guangzhou, China). Cells were transfected with siRNAs using Lipofectamine 2000 (Invitrogen, Life Technologies Co.).

### GSIS Assay

Isolated mouse islets were seeded into 250 µl of RPMI-1640 medium with 11.1 mmol/l glucose in 48-well dishes at 8 islets/well; Min-6 cells were seeded into 250 µl RPMI-1640 medium with 11.1 mmol/l glucose at 1×10^5^ cells/well in 48-well dishes, then cultured and treated under several conditions. Following preincubation for 1 h in KRB buffer containing 3.3 mmol/l glucose, the islets were treated for 1 h in KRB buffer and drug solutions with low (3.3 mmol/l glucose) or stimulatory (16.7 mmol/l glucose) concentrations of glucose. The supernatants were then obtained from each reaction well and frozen at −70°C for subsequent determination of insulin concentration. The insulin levels were measured using a radioimmunoassay as described previously [31].

### MTT Assay

Cell viability was determined using an MTT assay (Sigma-Aldrich Co.). Briefly, the cells were seeded in 96-well dishes at 1×10^4^ to 2×10^4^ cells per well, and treated with or without mCPP for 12 h. Then each well was supplemented with 10 µl MTT and incubated for 4 h at 37°C. The medium was then removed and 150 µl of DMSO (Sigma-Aldrich Co.) was added to solubilize the MTT formazan. For quantification, the optical density was read at 490 nm.

### Statistical Analysis

Statistical analysis was performed with SPSS 11.0 software (SPSS Inc., Chicago, IL). Comparisons were performed using Student’s *t* test between two groups, or ANOVA in multiple groups. Results are presented as means ± SEM. A *P*-value <0.05 was considered to be a statistically significant difference.

## Results

### Expression of 5-HT_2C_R in Pancreatic Islets of db/db Mice

**Figure 1:**
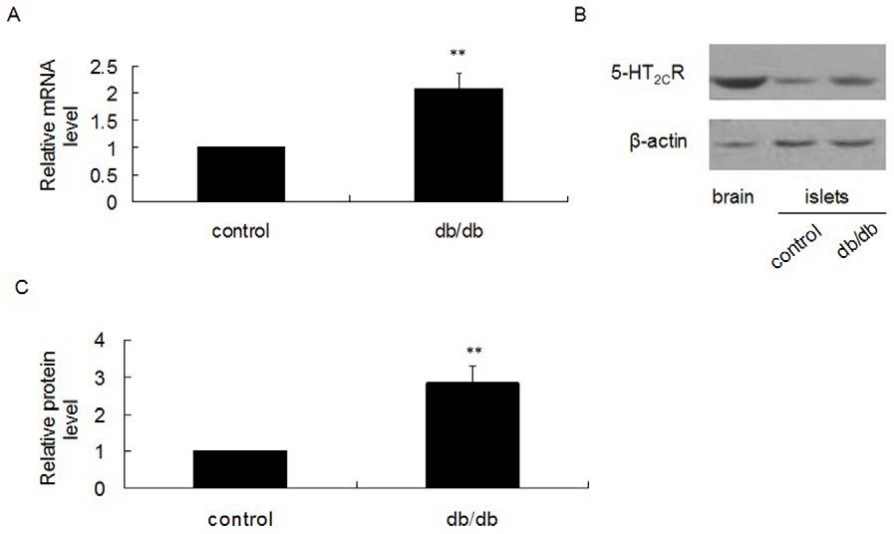
Expression of 5-HT_2C_R in pancreatic islets of db/db mice. A: mRNA level of 5-HT_2C_R was significantly higher in pancreatic islets of db/db mice compared to control mice. B: Protein level of 5-HT_2C_R was significantly higher in pancreatic islets of db/db mice compared to control mice. C: Relative protein level of 5-HT_2C_R. Graphs are shown as a ratio of 5-HT_2C_R to β-actin and compared to controls. All bands were normalized as percentages of the control values. Shown are representative results (average of duplicates) of at least three independent experiments. (^**^ *P*<0.01).

**Figure 2:**
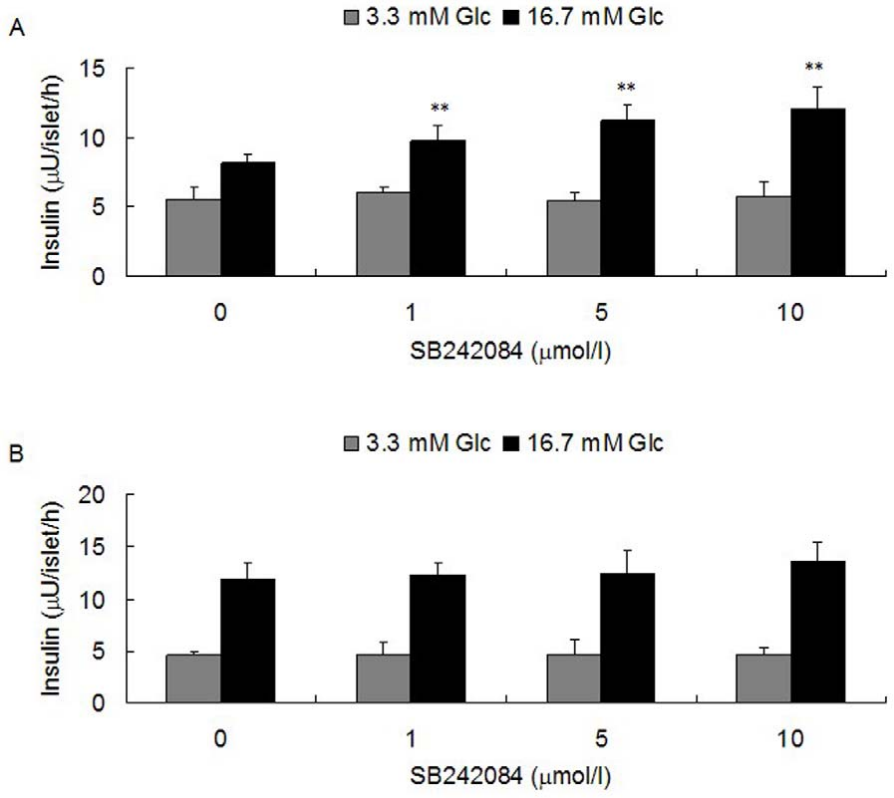
Effect of SB242084 on insulin secretion from pancreatic islets of control and db/db mice. After treatment with 1 to 10 µmol/l of SB242084 for 12 h, insulin secretion increased in pancreatic islets of db/db mice (A), but not in pancreatic islets of control mice (B; islets per well = 8; wells per group = 6; ^**^ *P*<0.01).

The db/db mouse line is a widely used animal model of T2DM, showing insulin resistance and impaired insulin secretion. In order to study the expression of 5-HT_2C_R in pancreatic islets of diabetic mice, we used 17 week old male db/db mice. Our data indicate that db/db mice have a higher body weight (***Fig. S1A***) and higher random blood glucose level (***Fig. S1B***) than control mice. We isolated pancreatic islets from db/db mice and control mice, and after overnight culture the total RNA and protein were extracted for analysis. Results from real-time quantitative PCR demonstrated that the mRNA level of 5-HT_2C_R was much higher in pancreatic islets isolated from db/db mice, compared to control mice (***Fig. 1A***). This result was confirmed by Western blot, which showed that the protein level of 5-HT_2C_R was also higher in pancreatic islets of db/db mice compared to control mice (***Fig. 1B***). Next, we investigated whether the higher expression level of 5-HT_2C_R could affect insulin secretion from pancreatic islets. We used 1, 5 and 10 µmol/l of the 5-HT_2C_R antagonist SB242084 to treat isolated pancreatic islets of db/db mice and control mice for 12 h, and then performed a GSIS test. We found that SB242084 treatment could improve insulin secretion from pancreatic islets isolated from db/db mice in a dose-dependent manner (***Fig. 2A***), but SB242084 had no significant effect on pancreatic islets from control mice (***Fig. 2B***).

### mCPP Decreased Insulin Secretion of Min-6 Cells and Mice Pancreatic Islets, which could be Reversed by SB242084

**Figure 3:**
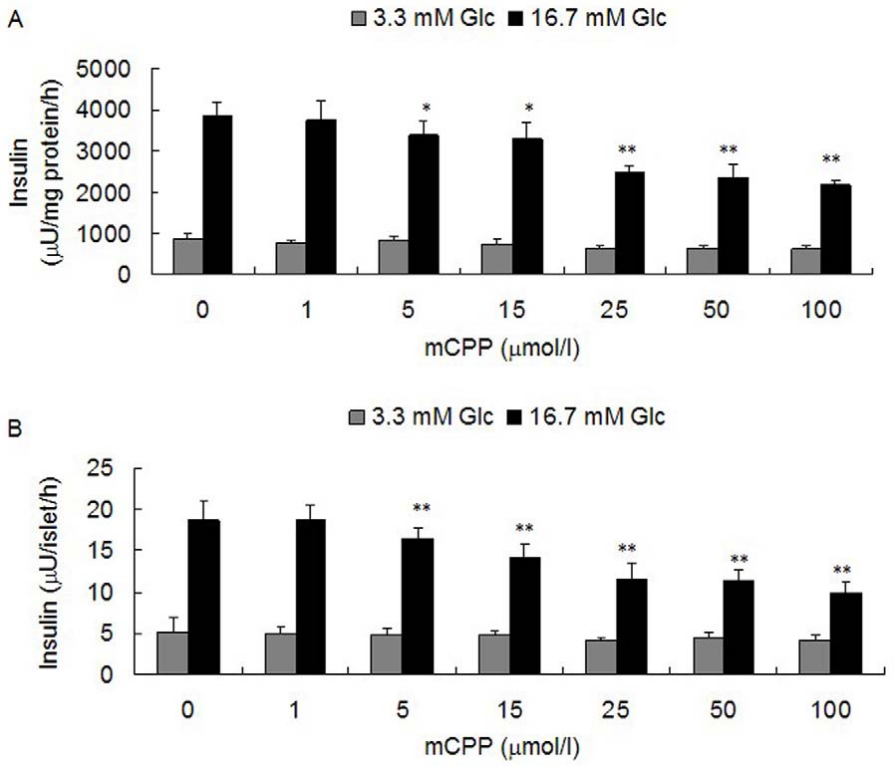
Effect of mCPP on insulin secretion from pancreatic β-cells. A: After treatment with 1 to 100 µmol/l mCPP for 12 h, insulin secretion from Min-6 cells under stimulus of 16.7 mM glucose decreased in a mCPP dose-dependent manner (n = 6; ^*^ *P*<0.05; ^**^ *P*<0.01). B: After treatment with 1 to 100 µmol/l mCPP for 12 h, insulin secretion from isolated mouse pancreatic islets under stimulus of 16.7 mM glucose decreased in a mCPP dose-dependent manner (islets per well = 8; wells per group = 6; ^*^ *P*<0.05; ^**^ *P*<0.01).

**Figure 4:**
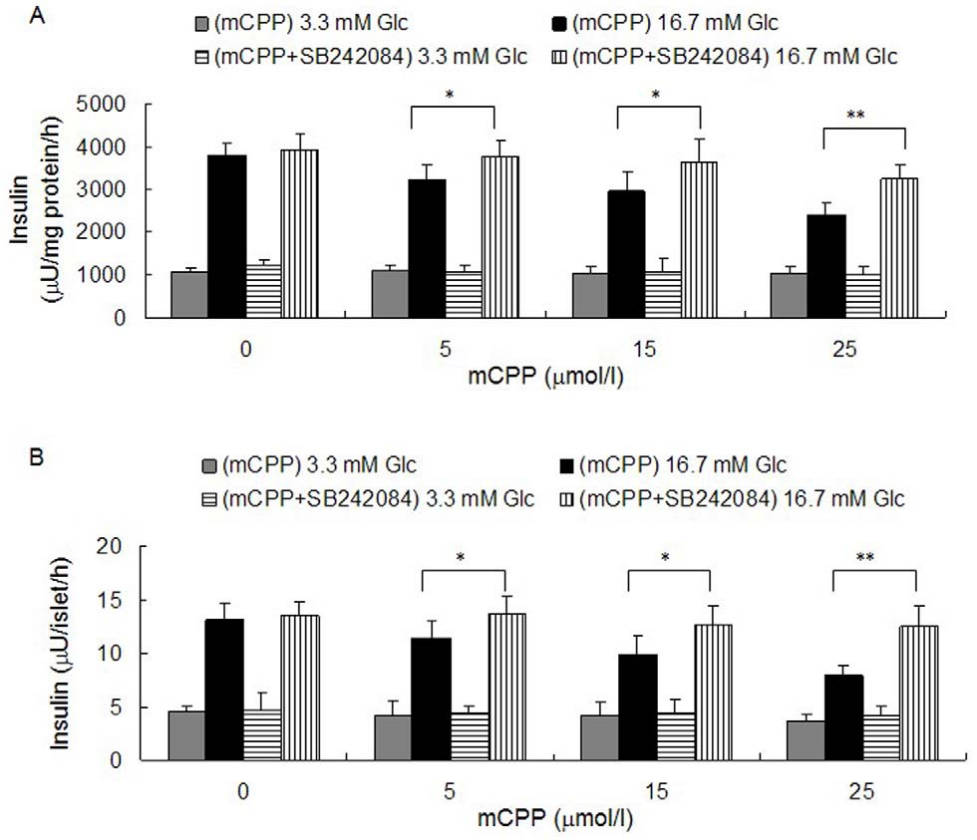
Effect of SB242084 on mCPP-induced inhibition of insulin secretion from pancreatic β-cells. A: For Min-6 cells, after treatment with 5 to 25 µmol/l mCPP with or without 5 µmol/l SB242084 for 12 h, the groups with SB242085 added showed higher insulin secretion under stimulus of 16.7 mM glucose, compared to control (n = 6; ^*^ *P*<0.05; ^**^ *P*<0.01). B: For isolated mouse pancreatic islets, after treatment with 5 to 25 µmol/l mCPP with or without 5 µmol/l SB242084 for 12 h, the groups with SB242085 added showed higher insulin secretion under stimulus of 16.7 mM glucose, compared to control (islets per well = 8; wells per group = 6; ^*^ *P*<0.05; ^**^ *P*<0.01).

One limitation to using db/db mice is that they are genetically modified animals, which might not fully represent normal physiology. In order to further study the effect of 5-HT_2C_R signaling on insulin secretion from pancreatic β-cells we used 1, 5, 15, 25, 50 and 100 µmol/l doses of the 5-HT_2C_R agonist mCPP to treat pancreatic islets isolated from ICR mice, as well as treating the pancreatic β-cell line Min-6 cells, for 12 h followed by a GSIS test. The results demonstrated that mCPP could decrease insulin secretion from both Min-6 cells (***Fig. 3A***) and isolated mouse pancreatic islets (***Fig. 3B***) in a dose-dependent manner. Five micromolar mCPP began to inhibit insulin secretion of pancreatic β-cells, and at 25 µmol/l mCPP was close to reach its maximum effect. The inhibitory effect of mCPP on insulin secretion of pancreatic β-cells in our study is in accordance with published in vivo data [26]. Considering the potential effect of mCPP on cell viability, we used a MTT assay to study whether mCPP had an effect on viability of Min-6 cells. After treatment with 1 to 100 µmol/l of mCPP for 12 h, Min-6 cells were assayed with MTT. The results showed that mCPP did not affect cell viability of Min-6 cells at any concentration (***Fig. S2***), which suggested a direct effect of mCPP on insulin secretion. In order to confirm that the effect of mCPP on insulin secretion was through activating 5-HT_2C_R, we used coadministration of 5, 15, or 25 µmol/l mCPP with 5 µmol/l SB242084 in Min-6 cells and isolated mouse pancreatic islets for 12 h, before carrying out a GSIS test. The results showed that SB242084 could significantly reverse the inhibitory effect of mCPP on insulin secretion both in Min-6 cells (***Fig. 4A***) and isolated mouse pancreatic islets (***Fig. 4B***).

### RNA Interference of 5-HT_2C_R of Min-6 Cells Reversed the Effect of mCPP on Insulin Secretion

**Figure 5:**
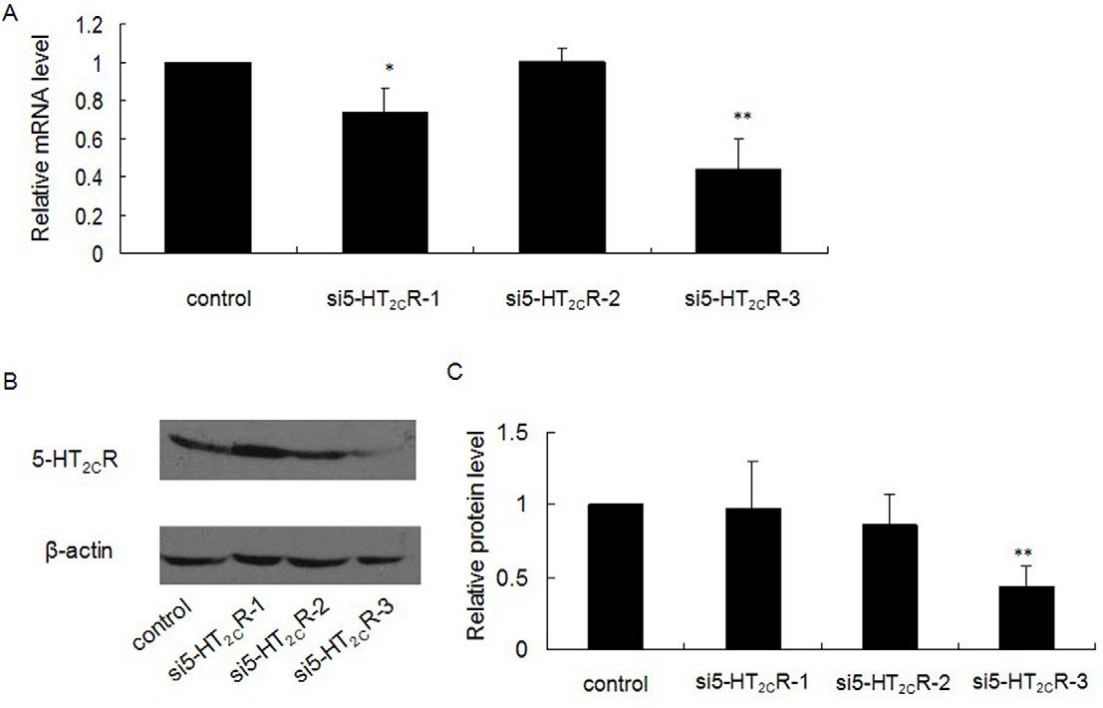
Screening of siRNA efficacy. Min-6 cells were transfected with si5-HT_2C_R-1, si5-HT_2C_R-2, si5-HT_2C_R-3 and control siRNA separately. After 48 h in culture expression of 5-HT_2C_R in each group was analyzed at the mRNA (A) and protein level (B). C: Relative protein level of 5-HT_2C_R. Graphs are shown as a ratio of 5-HT_2C_R to β-actin and compared to controls. All bands were normalized as percentages of the control values. Shown are representative results (average of duplicates) of at least three independent experiments. (^*^ *P*<0.05; ^**^ *P*<0.01).

**Figure 6:**
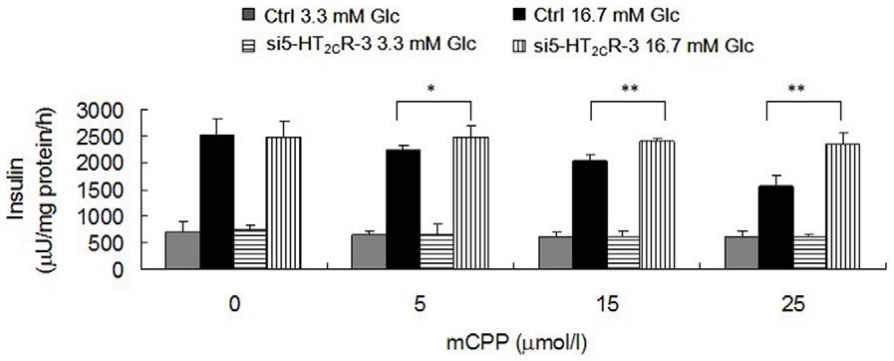
Effect of RNA interference of 5-HT_2C_R on mCPP-induced inhibition of insulin secretion from Min-6 cells. Min-6 cells were transfected with si5-HT_2C_R-3 or control siRNA. After 36 h in culture cells were treated with 5, 15 or 25 µmol/l mCPP for another 12 h. The si5-HT_2C_R-3-treated groups showed higher insulin secretion under stimulus of 16.7 mM glucose compared to control (n = 6; ^*^ *P*<0.05; ^**^ *P*<0.01).

To further investigate the role of 5-HT_2C_R and the inhibitory effect of mCPP on insulin secretion from pancreatic β-cells, we used RNA interference against the 5-HT_2C_R in Min-6 cells. Transfection of Min-6 cells with si5-HT_2C_R-1, si5-HT_2C_R-2, si5-HT_2C_R-3 and control siRNA was carried out separately. After culturing for 48 h in the presence of the siRNA, we analyzed the expression of 5-HT_2C_R in each group. We found that si5-HT_2C_R-3 could significantly decrease the expression of 5-HT_2C_R at the mRNA (***Fig. 5A***) and protein level (***Fig. 5B***). As such, further experiments were carried out with this siRNA. After transfection of Min-6 cells with si5-HT_2C_R-3 and 36 h in culture, we added 5, 15, or 25 µmol/l mCPP to treat the cells for 12 h, and then performed a GSIS test. The results showed that RNA interference of 5-HT_2C_R could reverse the inhibitory effect of mCPP on insulin secretion in Min-6 cells (***Fig. 6***), which was in accordance with the effect of SB242084.

### Palmitic Acid Increases the Expression of 5-HT_2C_R in Min-6 Cells

**Figure 7:**
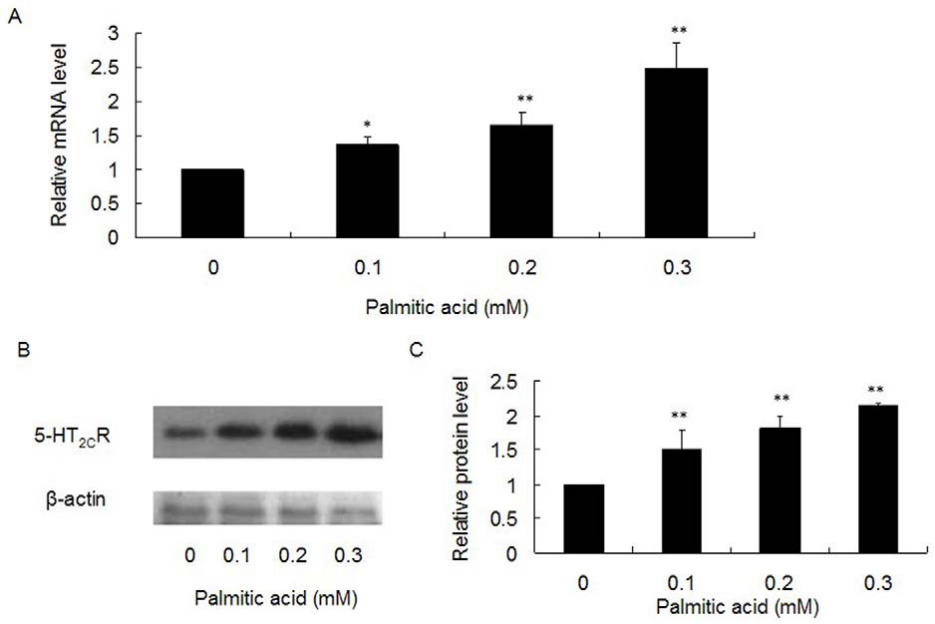
Effect of palmitic acid on expression of 5-HT_2C_R in Min-6 cells. Min-6 cells were treated with 0.1, 0.2 or 0.3 mM of palmitic acid for 24 h, and then the expression of 5-HT_2C_R was analyzed at the mRNA (A) and protein level (B). C: Relative protein level of 5-HT_2C_R. Graphs are shown as a ratio of 5-HT_2C_R to β-actin and compared to controls. All bands were normalized as percentages of the control values. Shown are representative results (average of duplicates) of at least three independent experiments. (^*^ *P*<0.05; ^**^ *P*<0.01).

Patients with T2DM and obese people usually have hyperlipidemia, which can cause insulin resistance and impaired insulin secretion [32]. There is evidence that excess free lipids in the plasma could generate neural toxicity, inducing altered neurotransmitter levels and/or abnormal function of their receptors, resulting in neural dysfunction, such as Alzheimer disease [33]. Furthermore, excess free lipids in plasma has also been shown to stimulate thrombocytes to secrete more 5-HT, leading to a high plasma concentration of 5-HT [34]. Considering the possible effect of elevated free lipids on the 5-HT system of pancreatic islets, we investigated the effect of palmitic acid on the expression of 5-HT_2C_R in pancreatic β-cells. We used 0.1, 0.2 and 0.3 mM concentrations of palmitic acid to treat Min-6 cells for 24 h, and then analyzed the effect on expression of 5-HT_2C_R. We found that at both the mRNA level (***Fig. 7A***) and protein level (***Fig. 7B***) of 5-HT_2C_R was increased in Min-6 cells following palmitic acid exposure in a dose-dependent manner. For example, in the 0.3 mM palmitic acid group, the mRNA level of 5-HT_2C_R increased more than 2-fold, with a further elevation of the protein expression level.

### SB242084 and RNA Interference of 5-HT_2C_R Improved Insulin Secretion of Min-6 Cells Treated with Palmitic Acid

**Figure 8:**
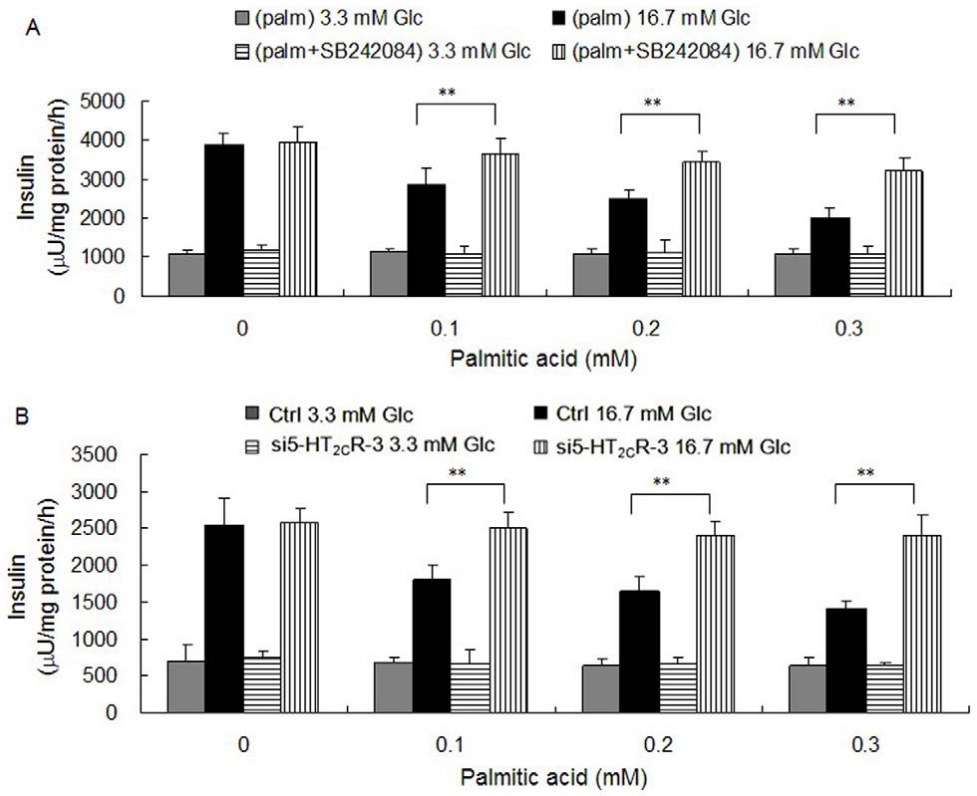
A: Effect of SB242084 on palmitic acid’s inhibition of insulin secretion from Min-6 cells. Min-6 cells were treated with 0.1 to 0.3 mM palmitic acid for 12 h, and were then treated with vehicle or 5 µmol/l SB242084 for a further 12 h. The groups with SB242084 added showed higher insulin secretion under stimulus of 16.7 mM glucose, compared to control (n = 6; ^**^ *P*<0.01). B: Effect of RNA interference of 5-HT_2C_R on palmitic acid mediated inhibition of insulin secretion from Min-6 cells. Min-6 cells were transfected with si5-HT_2C_R-3 or control siRNA and cultured for 24 h, next 0.1 to 0.3 mM palmitic acid was added for a further 24 h. The si5-HT_2C_R-3-treated groups showed higher insulin secretion under stimulus of 16.7 mM glucose compared to control (n = 6; ^**^ *P*<0.01).

Next we investigated whether the increased expression of 5-HT_2C_R stimulated by palmitic acid in Min-6 cells was related to the deleterious effects of palmitic acid on insulin secretion. We used 0.1, 0.2 and 0.3 mM concentrations of palmitic acid to treat Min-6 cells for 12 h, and then added 5 µmol/l SB242084 for a further 12 h before performing a GSIS test. Our results show that palmitic acid had a deleterious effect on insulin secretion from Min-6 cells, with an approximately 65% decrease in insulin secretion at a concentration of 0.3 mM palmitic acid; however, addition of 5 µmol/l SB242084 lead to a higher level of insulin secretion than the control groups (***Fig. 8A***), demonstrating an improvement on the function of Min-6 cells even in such cytotoxic circumstances. RNA interference of 5-HT_2C_R expression in Min-6 cells was next used to study their ability to secrete insulin after treatment with palmitic acid. We transfected Min-6 cells with si5-HT_2C_R-3 and cultured then for 24 h. Next we added 0.1, 0.2, or 0.3 mM palmitic acid for a further 24 h, and then performed a GSIS test. We found that RNA interference of 5-HT_2C_R could improve insulin secretion from Min-6 cells after treated with palmitic acid (***Fig. 8B***), in accordance with the results from our experiments with SB242084.

### Expression of tph in Pancreatic Islets of db/db Mice and Min-6 Cells Treated with Palmitic Acid

**Figure 9:**
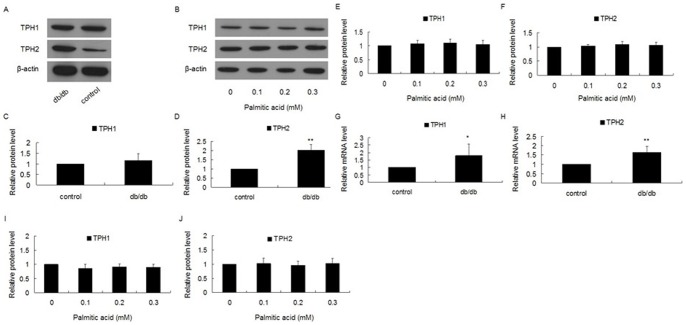
A: Protein level of TPH in pancreatic islets of db/db mice compared to control mice. B: Effect of palmitic acid on protein level of TPH in Min-6 cells. Min-6 cells were treated with 0.1, 0.2 or 0.3 mM of palmitic acid for 24 h, and then the expression of TPH were analyzed. C: Relative protein level of TPH1 in (A). D: Relative protein level of TPH2 in (A). E: Relative protein level of TPH1 in (B). F: Relative protein level of TPH2 in (B). G: mRNA level of TPH1 was significantly higher in pancreatic islets of db/db mice compared to control mice. H: mRNA level of TPH2 was significantly higher in pancreatic islets of db/db mice compared to control mice. I: Effect of palmitic acid on mRNA level of TPH1 in Min-6 cells. J: Effect of palmitic acid on mRNA level of TPH2 in Min-6 cells. (^*^ *P*<0.05, ^**^ *P*<0.01).

5-HT is synthesised in two steps from the essential amino acid tryptophan, which is acquired in the diet. Tryptophan is first hydroxylated at the 5 position of the indole ring by tryptophan hydroxylase (TPH), yielding 5-hydroxytryptophan; this product is then decarboxylated by aromatic L-amino acid decarboxylase, yielding 5-HT. Tryptophan hydroxylase is the rate-limiting enzyme in 5-HT synthesis [35]. There are two isoforms of tryptophan hydroxylase in pancreatic β-cells: TPH1 and TPH2 [36]. And it is reported that expression of both isoforms of TPH increased in β-cells in some cases, such as pregnancy, leading to high content of 5-HT [36]. So we investigated the expression of TPH1 and TPH2 in pancreatic islets of db/db mice and Min-6 cells treated with palmitic acid. We found that in islets of db/db mice, the mRNA level of TPH1 increased significantly (***Fig. 9G***), but the change of protein level did not reach the statistical significance (***Fig. 9A***). Both mRNA level and protein level of TPH2 significantly increased than control mice (***Fig. 9H*** ***;9A***). Then we used 0.1, 0.2 and 0.3 mM concentrations of palmitic acid to treat Min-6 cells for 24 h, and then analyzed the effect on expression of TPH1 and TPH2. Interestingly, expression of TPH1 (***Fig. 9B*** ***;9I***) and TPH2 (***Fig. 9B*** ***;9J***) did not change in Min-6 cells after treatment with palmitic acid.

## Discussion

T2DM is a metabolic disease characterized by elevated blood glucose levels; both environmental and genetic factors can lead to the development of diabetes [37]. Due to changes in food structure and lifestyle, the global incidence of T2DM has become much higher [38]. In addition to insulin resistance and impaired function of pancreatic β-cells in patients with T2DM [39], [40], this study focused on insulin secretion affected by 5-HT_2C_R, which has been suggested the possibility that an abnormal 5-HT system could also affect regulation of energy metabolism [41].

In our study, we found that expression of the 5-HT_2C_R was much higher in pancreatic islets of db/db mice than control mice, which was in accordance with the higher 5-HT_2C_R level reported in the hypothalamus of obese Ay mice [42]. We used the 5-HT_2C_R antagonist SB242084 to study whether the inhibition of 5-HT_2C_R could affect insulin secretion from pancreatic islets, finding that after treatment with SB242084, pancreatic islets isolated from db/db mice had improved insulin secretion in a SB242084 dose-dependent manner. Interestingly, pancreatic islets from control mice were also weakly affected by SB242084, though this was without statistical significance. In order to further study the effect of 5-HT_2C_R on the function of pancreatic β-cells, we used the 5-HT_2C_R agonist mCPP to treat Min-6 cells and isolated pancreatic islets for 12 h, finding that insulin secretion from both Min-6 cells and isolated pancreatic islets was significantly decreased in a mCPP dose-dependent manner, with no effect on cell viability or insulin content (***Fig. S3***). This inhibitory effect of mCPP on insulin secretion from pancreatic β-cells could be reversed by treatment with SB242084 or RNA interference of 5-HT_2C_R, which demonstrated that 5-HT_2C_R played an inhibitory role in insulin secretion from pancreatic β-cells. We had also taken other 5-HT receptors into consideration. In our preliminary experiments, we examined the expression of 5-HT receptors 2A, 2B, 2C, 1A, 1B in mice islets and Min-6 cells with RT-PCR, but we could not detect other 5-HT receptors except for 5-HT_2C_R (***Fig. S4***). We also examined whether 1 µmol/l SB242084 could reverse the inhibitory effect of 5 to 25 µmol/l mCPP on insulin secretion in Min-6 cells in our preliminary experiments, but we found that 1 µmol/l SB242084 could not work well when the concentration of mCPP was 25 µmol/l (***Fig. S5***), which may suggest insufficient dosage of SB242084. It has been reported that activation of 5-HT_2C_R could inhibit the firing rate of dopaminergic neurons and reduce dopamin release [43], [44], [45]. The firing rate is also critical for pancreatic β-cells to secret insulin [46]. The 5-HT_2C_R may decrease insulin secretion through inhibiting firing rate of β-cells, and could also affect β-cell membrane capacitance/voltage-fated calcium current, docked granule pools, SNARE complex, etc. All these potential mechanism and which secretion phase was affected need further research.

Considering the negative effect of excess free lipids on the function of pancreatic β-cells, we investigated whether 5-HT_2C_R facilitated the deleterious effect of palmitic acid on Min-6 cells. We found that after treatment with palmitic acid for 24 h, Min-6 cells expressed higher levels 5-HT_2C_R in a dose-dependent manner, suggesting a possible role for 5-HT_2C_R in the deleterious effect of palmitic acid on insulin secretion from Min-6 cells. Subsequent experiments demonstrated that increased expression of 5-HT_2C_R partly induced the inhibitory effects of palmitic acid on insulin secretion from Min-6 cells, which could be reversed by SB242084 or RNA interference of 5-HT_2C_R.

At last, we investigated the expression both isoforms of TPH in islets of db/db mice and Min-6 cells treated with palmitic acid. The higher expression of tph2 in islets of db/db mice may reveal higher content of 5-HT. Interestingly, we did not observe any change of expression of TPH1 and TPH2 in Min-6 cells after treatment with different concentration of palmitic acid. But it has been reported that unsaturated fatty acids could induce 5-HT release from platelets [47] and the fish oil will affect 5-HT turnover in hypothalamus [48]. The effect of fatty acids on 5-HT system may largely rely on 5-HT release. Anyway, the serotonin biosynthesis and release was not measured in islets or Min-6 cells, it is still unclear whether 5-HT_2C_R mediates the effect of serotonin release by neuron synapses and/or via β-cells in vivo, and the affect of high glucose plus high fatty acids to TPH expression in pancreatic β-cells should be done in future study.

Our results might help to explain the delayed elevation of postprandial plasma insulin level in T2DM patients. Though this phenomenon has a close relationship to incretins [49], we believe the action of the incretins does not entirely mediate this effect. In our working model, T2DM patients have high expression levels of 5-HT_2C_R in pancreatic β-cells, thus, after meal ingestion the intrapancreatic serotonergic nerves secrete 5-HT around pancreatic islets, which could activate the 5-HT_2C_R of pancreatic β-cells, resulting in decreased insulin secretion. Anyway, such a phenomenon could also be recognized as the result of some kind of protective physiological changes in T2DM patients or obese people. That is to say, when the body has excess energy storage, it could up regulate the expression of 5-HT_2C_R in hypothalamus to drive the anorexic effect [50], and could also up regulate the expression of 5-HT_2C_R in pancreatic β-cells resulting in less insulin secretion after meals, leading to less energy intake from food ingestion. Increased expression of 5-HT_2C_R in both hypothalamus and β-cells could mediate this protective strategy to prevent excess energy intake. Moreover, in evolutionary terms the presence of 5-HT synthesis in plants [51] as well as all branches of metazoan life [52], [53] demonstrates that the 5-HT system arose relatively early in the evolution of life. This indicates that the 5-HT system evolved before the plant–animal evolutionary divergence, which was estimated to have occurred 1.5 billion years ago. Functioning as a trophic factor in plants, 5-HT signaled in even the most primitive nervous systems to regulate the primitive energy metabolism systems [52], [53]. Considering that pancreatic islet cells and neurons share common functions and similar ontogenies [54], it is not surprising that the serotonergic nervous system might regulate pancreatic islet function, to form an intricate energy metabolism regulatory system with the effect on hypothalamus. Our data strongly suggest that the 5-HT system is important for metabolic control, though much remains to be understood about the function of the 5-HT system in energy metabolism, including the specific roles of each of the 5-HT receptor subtypes, and the nuances of the effector pathways. In summary, our data demonstates that 5-HT_2C_R might play a role in the dysfunction of pancreatic β-cells in T2DM patients, this novel finding brings a new understanding of T2DM etiology, and may provide new avenues to treat this disease.

## Supporting Information

Figure S1 **Body weight, random blood glucose of db/db mice and control mice.** A: Body weight of db/db mice was higher than control mice. B: Random blood glucose of db/db mice was higher than control mice (n = 6; ^**^ *P*<0.01).
(TIF)

Figure S2 **Effect of mCPP on viability of Min-6 cells.** After treatment with 1 to 100 µmol/l mCPP for 12 h, cell viability of Min-6 cells was analyzed by MTT assay. No difference was seen between the mCPP-treated groups and control cells. (n = 4).
(TIF)

Figure S3 **Effect of mCPP on insulin content of pancreatic β-cells.** A: After treatment with 1 to 100 µmol/l mCPP for 12 h, insulin content of Min-6 cells were analyzed (n = 6). B: After treatment with 1 to 100 µmol/l mCPP for 12 h, insulin content of isolated mouse pancreatic islets were analyzed (islets per well = 8; wells per group = 6).
(TIF)

Figure S4 **Analysis of 5-HTR 1A, 1B, 2A, 2B, 2C in Min-6 cells and mouse islets with RT-PCR, only 5-HT_2C_R was detectable.**
(TIF)

Figure S5 **Effect of 1 µmol/l SB242084 on mCPP-induced inhibition of insulin secretion from mouse pancreatic islets.** After treatment with 5 to 25 µmol/l mCPP with or without 1 µmol/l SB242084 for 12 h, the groups with SB242085 added showed higher insulin secretion under stimulus of 16.7 mM glucose in 5 to 15 µmol/l mCPP groups, compared to control (islets per well = 8; wells per group = 6; ^*^ *P*<0.05).
(TIF)
