# Supplementary figures and images for: Serotonin Receptor 2C and Insulin Secretion
Source: PLoS One. 2013 Jan 17;8(1):e54250. doi: 10.1371/journal.pone.0054250 (PMC3547871; doi:10.1371/journal.pone.0054250)

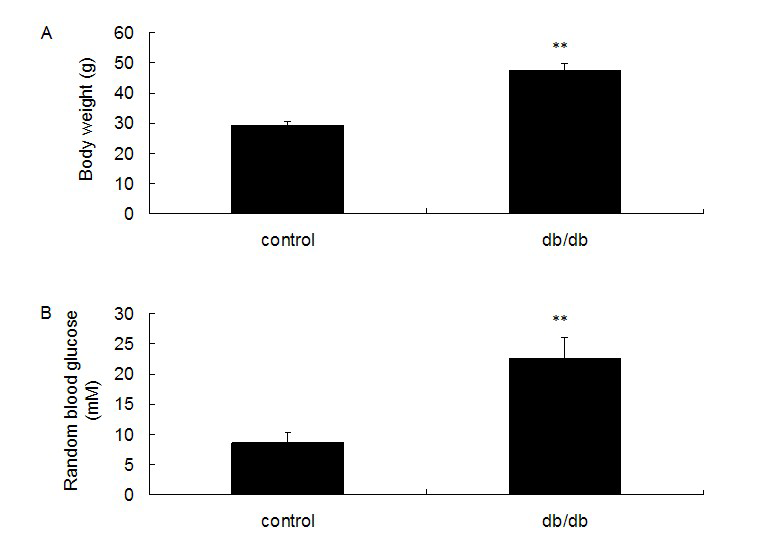

Supplement: Figure S1 — Body weight, random blood glucose of db/db mice and control mice. A: Body weight of db/db mice was higher than control mice. B: Random blood glucose of db/db mice was higher than control mice (n = 6; ** P<0.01). (TIF) [file pone.0054250.s001.tif]

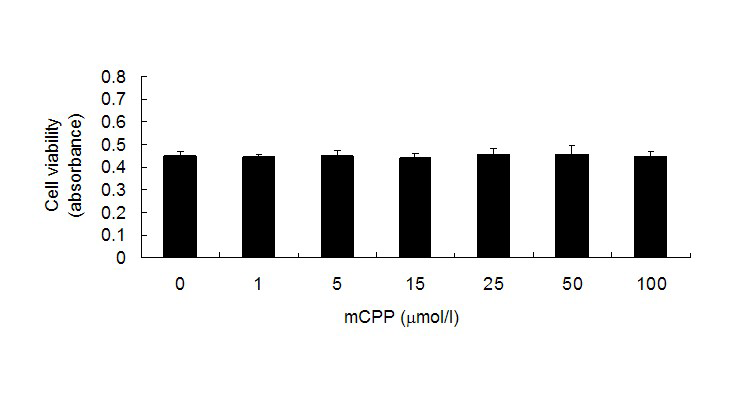

Supplement: Figure S2 — Effect of mCPP on viability of Min-6 cells. After treatment with 1 to 100 µmol/l mCPP for 12 h, cell viability of Min-6 cells was analyzed by MTT assay. No difference was seen between the mCPP-treated groups and control cells. (n = 4). (TIF) [file pone.0054250.s002.tif]

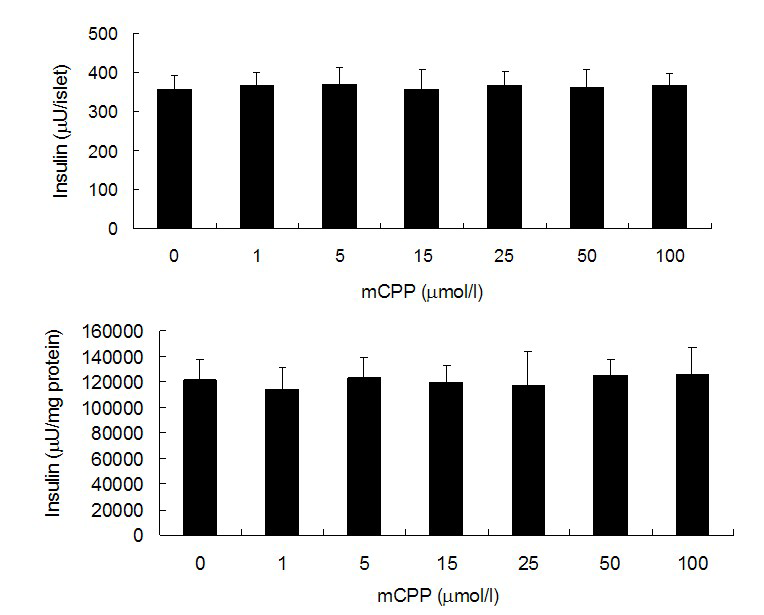

Supplement: Figure S3 — Effect of mCPP on insulin content of pancreatic β-cells. A: After treatment with 1 to 100 µmol/l mCPP for 12 h, insulin content of Min-6 cells were analyzed (n = 6). B: After treatment with 1 to 100 µmol/l mCPP for 12 h, insulin content of isolated mouse pancreatic islets were analyzed (islets per well = 8; wells per group = 6). (TIF) [file pone.0054250.s003.tif]

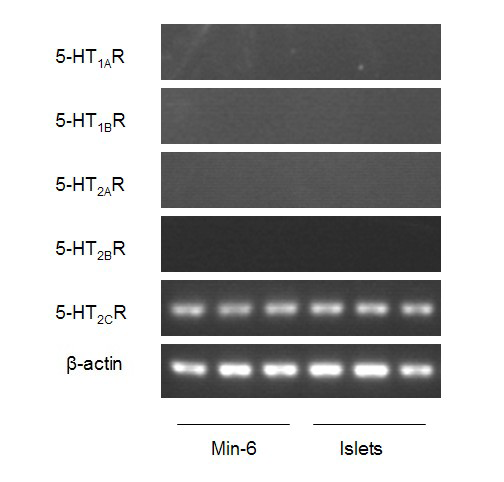

Supplement: Figure S4 — Analysis of 5-HTR 1A, 1B, 2A, 2B, 2C in Min-6 cells and mouse islets with RT-PCR, only 5-HT2CR was detectable. (TIF) [file pone.0054250.s004.tif]

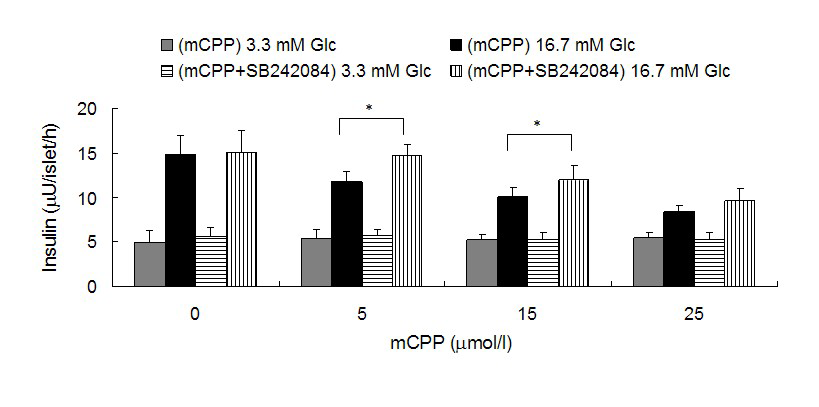

Supplement: Figure S5 — Effect of 1 µmol/l SB242084 on mCPP-induced inhibition of insulin secretion from mouse pancreatic islets. After treatment with 5 to 25 µmol/l mCPP with or without 1 µmol/l SB242084 for 12 h, the groups with SB242085 added showed higher insulin secretion under stimulus of 16.7 mM glucose in 5 to 15 µmol/l mCPP groups, compared to control (islets per well = 8; wells per group = 6; * P<0.05). (TIF) [file pone.0054250.s005.tif]
